# Child and adolescent mental health services in Uganda

**DOI:** 10.1186/s13033-021-00491-x

**Published:** 2021-08-03

**Authors:** Silje Akselberg Iversen, Joyce Nalugya, Juliet N. Babirye, Ingunn Marie Stadskleiv Engebretsen, Norbert Skokauskas

**Affiliations:** 1 Faculty of Medicine and Health Sciences, NTNU, Trondheim, Norway; 2 Department of Psychiatry, Makerere University College of Health Sciences, Kampala, Uganda; 3 Makerere University School of Public Health, Kampala, Uganda; 4 Center for International Health, Department of Global Health and Social Medicine, Faculty of Medicine, University of Bergen, Bergen, Norway; 5 Regional Knowledge Center for Children and Adolescents – Mental Health and Child Welfare, IPH; NTNU, FMH, Trondheim, Norway

**Keywords:** Uganda, Mental health, Mental disorders, Child and adolescent, Child and adolescent mental health services (CAMHS)

## Abstract

**Introduction:**

Worldwide, one in five children and adolescents suffer from mental health disorders, while facing limited opportunities for treatment and recovery. Growing up, they face multiple challenges that might contribute to the development of mental disorders. Uganda is a developing country with a history of prolonged civil and regional wars associated with child soldiers, large numbers of refugees and internally displaced people due to natural disasters and unrests, and a large infectious disease burden mainly due to acute respiratory tract infections, malaria and HIV/AIDS.

**Objective:**

This paper aims to examine the current status of child and adolescent mental health services in Uganda.

**Methodology:**

A scoping review approach was used to select studies on child and adolescent mental health services (CAMHS) in Uganda. A search of MEDLINE, Wiley and PubMed databases was conducted using eligibility criteria. The papers were summarized in tables and then synthesized using the Frameworks for monitoring health systems performance designed by the World Health Organisation (WHO). This was done according to the Preferred Reporting Items for Systematic Review and M-Analyses Extension for Scoping Review (PRISMA-ScR) guidelines.

**Results:**

Twelve studies were identified; five of them used qualitative methods and focused mostly on the current limitations and strengths of CAMHS in Uganda, while six quantitative studies investigated the effects of new interventions. One study used a mixed-methods approach. In summary, the papers outlined a need for collaboration with the primary health sector and traditional healers to ensure additional human resources, as well as the need to focus on groups such as orphans, HIV/AIDS-affected youth, former child soldiers and refugees.

**Conclusion:**

Relatively few studies have been conducted on CAMHS in Uganda, and most of those that exist are part of larger studies involving multiple countries. CAMHS in Uganda require improvement and needs to focus especially on vulnerable groups such as orphans, HIV/AIDS-affected youth and former child soldiers.

## Background

Mental health is integral to overall health, as stated by the first Director-General of the World Health Organization (WHO), Dr. Brock Chisholm, in 1954: “without mental health, there can be no true physical health” (1). While mental health is a key element of WHO’s definition of health, there is a significant lack of funding and availability of mental health services, including Child and adolescent mental health services (CAMHS) that assess and treat young people with emotional, behavioral or mental health difficulties [1]. For example, in Uganda, there are only five child and adolescent psychiatrists for more than 20 million children and adolescents [2, 3]. Children and adolescents worldwide may also be exposed to stress, such as pressure from peers and at school or difficulties in accepting their identity and sexuality, as well as pressure from social media on issues such as body image, success and popularity [4, 5]. The stress they go through contributes to one in five children and adolescents worldwide suffering from mental illnesses, and access to treatment is insufficient and/or unevenly distributed [5]. Half of those with mental illness have symptoms before the age of 14, and early intervention is crucial to better outcomes [6]. Worldwide, there is therefore an urgent need for a scaling up of child and adolescent mental health services [7].

A large proportion of the Ugandan population are children and adolescents (57% are under 15 years), grappling with many of the issues outlined above; this is compounded by prevailing poverty, with 20% of the population living below the poverty line [3]. Uganda also has a history of prolonged armed conflicts, such as that in the north of the country involving the Lord’s Resistance Army which lasted about 20 years and resulted in 1.7 million children living without their parents from the various armed conflicts [8]. Orphans are more likely to have greater emotional needs, to be insecure and to live in poverty with elderly widowed female caregivers [8]. In addition to facing the regular challenges of childhood and adolescence, many young people in Uganda, therefore, have to combat additional challenges that contribute to a great burden of suffering [9, 8].

Moreover, the conflict with the Lord’s Resistance Army led not only to orphanhood but also to the abduction of children and adolescents to be used as child soldiers [10]. Even though this conflict has now ceased the consequences persist, as abducted children were forced to carry out raids and kill and mutilate others, and up to 97.7% of them have later experienced Post-traumatic stress disorder (PTSD) [8]. Among these children, more than one-third of abducted girls were also sexually abused [8]. Another issue is that former child soldiers and their families face discrimination and social stigma due to their association with the Lord’s Resistance Army, adding to the risk of them developing mental disorders [5, 8, 10]. Early intervention could prevent or reduce both current and future mental health symptoms [11].

Uganda has made remarkable strides in peaceful development and reducing poverty over recent years, and this has translated into greater investment in health care overall. However, funding remains low by international standards; for example as of 2019 only 9.8% of Gross Domestic Product (GDP) was allocated to health and only 1% of GDP was allocated for mental health services [12]. The allocation to mental health care includes services for adults as well as children and adolescents [12]. This picture highlights the very low prioritization of mental health services in dispensing of resources in Uganda despite the adoption of the Child and Adolescent Mental Health Policy Guidelines in 2017 [13]. These guidelines were designed to promote Mental Health and prevent mental, neurological and substance use disorders among children and adolescents [13]. The specific objectives of these policy guidelines are [13]: (1) To increase knowledge, understanding and involvement of policy makers, service providers, families, community leaders and other stakeholders for the promotion of Mental Health and prevention of mental, neurological and substance use disorders in children and adolescents; (2) To build capacity, improve access and availability of comprehensive CAMHS for care and treatment of children and adolescents affected by them including psychological treatment and rehabilitation of such children; (3) To strengthen research, monitoring and evaluation of child and adolescent mental health; (4) To inform the development and review of policies and legislation relating to child and adolescent mental health; (5) To strengthen multi-sectoral collaboration and participation in providing quality CAMHS. The guidelines are intended for use by all government sectors and civil society stakeholders for a period of ten years following adoption in 2017 [13]. The Ugandan guidelines are not stand alone but are supported by global initiatives such as the WHO Mental Health Gap Action Programme (mhGAP) and mhGAP Intervention Guide (mhGAP-IG) which have attempted to address the substantial needs for mental health services. WHO calls for the mhGAP-IG to be implemented in both pre-service and in-service training, integrating mental health into primary health care in the long term [14]. Several studies have examined the current status of CAMHS in the country, but there is no general overview of the situation that would allow for recommendations for the future to be made. This paper aims to examine the current status of CAMHS in Uganda so as to inform implementation of the Ugandan Child and Adolescent Mental Health Policy Guidelines of 2017.

## Methodology

This study used a scoping review approach and employed the following eligibility criteria for papers to be included:

- Written in English
- Published between January 2000 and July 2020
- Countries of origin of study: Uganda
- Study design: randomized controlled trial, blind trial, non-blind trial, adaptive clinical trial, non-randomized trial, interrupted time series design, cohort study, case–control study and cross-sectional study.

Three databases (PubMed, MEDLINE, Wiley Online Library) were searched between August 2020 and October 2020 using the terms «mental health services», «psychiatry», «child and adolescent», «youth» and «Uganda». Titles and abstracts were examined using the inclusion criteria, after which full articles were retrieved. This was done according to Preferred Reporting Items for Systematic Review and M-Analyses Extension for Scoping Review (PRISMA-ScR) guidelines [15].

Two of the researchers reviewers (S.A.I., N.S:) extracted the relevant data from the included studies with a predefined data recording form, addressing the search criteria. Both researchers checked the completeness and accuracy of the data extraction for all included studies. To resolve discrepancies, other co-authors were consulted to help with consensus development. The following core data were extracted from all included studies: title, authors, and other publication details; study design and aim, setting (place and time of recruitment/data collection), study population characteristics (age, gender, and diagnostic criteria used, sample size etc.), intervention characteristics (type of medication used, duration of intervention); methods of outcome measurement, (statistical methods and results related to the outcomes).

## Results

### Study selection

The initial online search produced 185 articles. Following the screening of all titles 41 eligible papers were identified, and then nine duplicates were removed. Finally, 32 full papers were assessed, 20 papers were excluded as not meeting inclusion criteria and 12 papers were included in the review.

### General description

The majority of the research papers were qualitative (n = 5) or quantitative studies (n = 6), while n = 1 used a mixed-methodology approach.

Table 1 summarizes the key findings regarding the current challenges and limitations of CAMHS in Uganda.

**Table 1:** Summary of studies regarding child and adolescent mental health services in Uganda

Study	Study location	Objective	Methods	Main results
*A. Qualitative studies*				
Mugisha et al. 2020 [16]	Masaka district, Central Uganda	To explore health professionals’ perspectives on barriers to seeking treatment among orphan children and adolescents with HIV/AIDS and mental distress	Semi-structured interviews with 15 health service managers and staff	Barriers to seeking treatment identified: family factors (caregivers with low or no education, lack of sufficient food to support care and treatment, lack of transport to reach health services, failure to buy drugs, family fatigue); individual factors (lack of motivation/exhaustion, lack of knowledge); community factors (stigma, lack of willingness to seek treatment, community failure to support families, community violence); health systems-level factors (limited service capacity, overwhelming burden from other diseases, child- and adolescent-unfriendly services, lack of medication, understaffing, lack of integrated care)
Skylstad et al. 2019 [17]	Mbale district, Eastern Uganda	To explore parents’ perspectives regarding child mental health, from the recognition of symptoms to help-seeking	Focus group discussions with 74 parents of children younger than 10 years in both urban and rural communities	Parents and the formal health system evaluate and handle symptoms of mental illness differently More mental health awareness is required to encourage parents to seek help for their child Multiple providers, such as traditional healers, were sought after, due to there being multiple beliefs and explanations connected with mental health symptoms, causes and treatments Loss of social support structures in the community
Akol et al. 2018 [11]	Eastern Uganda	To explore traditional healers’ views on their collaboration with biomedical health systems	Semi structured in-depth interviews with 20 traditional healers with Child and Adolescent mental health experience	Traditional healers expressed distrust in biomedical health systems and believed that their treatments were superior. There is a need to increase collaboration between the traditional and biomedical sectors of mental health care to improve access to CAMHS Traditional healers should be able to recognize and refer children with mental health issues to CAMHS
Akol et al. 2015 [18]	Kampala and Mbale, Eastern Uganda	To explore strengths and weaknesses of CAMHS at national and district levels from a management perspective	Semi-structured interviews with 7 public officials responsible for management and supervision of CAMHS at national level (Kampala) and district level (Mbale)	Inadequate national mental health policies, inadequate Child and adolescent mental health financing and services; a solution is to integrate child and adolescent mental health into primary health care and other sectors CAMHS absent at lower-level health centres (primary health care centres) Insufficient CAMHS workforce should be increased by both in-service and pre-service training Health management information systems are insufficient for service planning
Okello et al. 2014 [19]	Northern and Central Uganda	To explore the mental health of young people in secondary schools in Northern and Central Uganda	Focus group discussions with 78 13–24-year-olds from 4 secondary schools, former child soldiers	There are key gaps in the knowledge and attitudes of young people (i.e., lack of knowledge about common mental health disorders, early signs of reduced functioning and poor mental health, and the link between substance use and mental illness) that need to be targeted by mental health interventions focused on young people
B. *Quantitative Studies*				
Kivumbi et al. 2019 [20]	Uganda	To examine the effect of participating in a family-based economic strengthening intervention (child development account, mentorship programme and workshops on financial management and microenterprise development) on the mental health wellbeing of female adolescent orphans impacted by HIV/AIDS in rural Uganda	Randomized trial consisting of female orphans aged 10–16 years. Intervention group (n = 516) received economic empowerment intervention in addition to standard care services for orphans, while control group (n = 273) received only standard care services	Improvement in mental health functioning over time among female participants receiving the economic empowerment intervention
Akol et al. 2018 [21]	Mbale and Sironko districts, Eastern Uganda	To evaluate the effect of primary health care provider mhGAP training on the identification and treatment of CAMH disorders	Randomized controlled trial. Intervention group (n = 18) clinics received mhGAP-oriented CAMH training based on the WHO mhGAP-IG (v1). Control group (n = 18 clinics) did not receive training	The training increased identification and reporting of non-epilepsy CAMH cases by primary health care clinics, but this increase did not reach statistical significance
Akol et al. 2017 [22]	Eastern Uganda	To describe an in-service CAMH (Child and adolescent mental health) training for non-specialist health workers in Uganda and assess cadre-differentiated learning outcomes To examine the possibility of integrating CAMH into primary health care to increase accessibility	Examined learning outcomes by pre- and post-training tests Intervention: 5 days of CAMH training for 36 non-specialist health workers	Increased CAMH knowledge for both nurses and clinical officers For the integration of CAMHS into primary health care, this kind of training should be offered
Ertl et al. 2011 [23]	Northern Uganda	To assess the efficacy of a community-based intervention targeting symptoms of PTSD in former child soldiers aged 12–25	Three randomized groups: narrative exposure therapy (n = 29), academic catch-up programme with elements of supportive counselling (n = 28), waiting list (n = 28)	Reduction of PTSD severity and other mental health problems (such as depression, etc.) especially in the narrative exposure therapy group, but also in the academic catch-up group
Han et al. 2013 [24]	Southwestern Uganda	To examine whether an innovative family economic empowerment intervention addresses the mental health functioning of AIDS-affected children in communities heavily impacted by HIV/AIDS	Randomized controlled trial among AIDS orphans in the last two years of primary school: intervention group (n = 179) received family economic empowerment interventions (promoting monetary savings for educational opportunities, financial management workshops, mentors) and the control group (n = 118) received standard aid (food aid, scholastic materials)	Children receiving the intervention reported significant improvement in their mental health functioning
Ssewamala et al. 2009 [25]	Uganda	To evaluate the effect of an economic empowerment intervention on health and mental health functioning among AIDS-orphaned adolescents	Randomized clinical trial consisting of AIDS orphaned children aged 11–17: intervention group (n = 131) received economic empowerment interventions (workshops, monthly mentorship programme, child development account), while control group (n = 137), received only usual care for orphaned children	The treatment group was over twice as likely as the control group to rate their health as good or excellent, using the Tennessee Self-Concept Scale to measure self-esteem and mental health wellbeing Due to the improvement in wellbeing in children and adolescents, including health and mental health functioning, which builds on the theory that positive links exist between assets and children’s wellbeing, there are implications for public policy and health programming for AIDS-orphaned adolescents, such as this economic empowerment intervention
*Mixed-methods studies*				
Kleintjes et al. 2010 [26]	Uganda, Ghana, South Africa and Zambia	To report on the findings of a situational analysis of CAMH policy and services in Ghana, Uganda, South Africa and Zambia To provide new knowledge regarding multisectoral approaches to breaking the cycle of poverty and mental ill-health in Africa	Quantitative study: WHO’s Assessment Instrument for Mental Health Systems (WHO-AIMS) Version 2.2 was used to collect information on mental health resources Qualitative study: focus group discussions (n = 13) and semi-structured interviews (n = 62) with public sector policy-makers and planners, nongovernmental programme managers, mental health care users, religious leaders and representatives of development agencies, professional associations and unions, university and research institutions	CAMH-related legislation, policies, services, programmes and human resources are scarce There is stigma around mental health, and it is given low priority, contributing to low levels of investment in CAMHS A lack of attention to the impoverishing impact of mental disorders on children and adolescents and their families contributes to the burden

Lastly, findings were summarized based on the six core components of the Frameworks for monitoring health systems performance designed by WHO, which is a tool to assess health systems through the key indicators of inputs, processes, outputs and outcome [15].

#### Health service delivery

This refers to the availability and distribution of health care, including factors such as comprehensiveness, accessibility, coverage, continuity, quality, person-centredness, coordination and accountability of the system [27]. One study cited limited service capacity, understaffing and burden from other diseases as an issue for the continuity and coverage of CAMHS, and reported that available services were not child- and adolescent-friendly and were absent at lower-level health centres (primary health care centres) [16]. Another barrier to the availability of CAMHS was that parents tend to turn to traditional healers first with their children, and such healers might not trust biomedical health systems and then choose to not refer children to CAMHS [17] (Table 1).

There is a lack of collaboration between CAMHS and other sectors within and outside the health system in Uganda, such as traditional healers and the primary health care sector [11]. CAMHS is also not yet integrated into primary health care, which could improve access to these services. This kind of collaboration should be sought after, but the training of primary health care professionals has yet to fully begin [18–22] (Table 1). This could give CAMHS increased continuity and accessibility, as described in the WHO frameworks [27].

Other studies called for collaboration between mental health treatment and HIV treatment, due to common co-occurrence, which creates a double burden of disease [18]. HIV-infected youth are often exposed to additional stressors such as stigma, orphanhood, poverty and neglect that make them vulnerable to developing mental health disorders. In addition, the stress that comes with being infected can itself affect their mental health negatively or worsen already present symptoms. However, these issues are rarely addressed in the country’s health systems; even though youth are receiving treatment for HIV, they should also receive support and counselling for mental health [16]. Another vulnerable group was found to be children and adolescents struggling with alcohol and substance use disorders, but they were rarely approached except for a few outreach activities in schools [18].

#### Health workforce (input)

Findings pointed towards a shortage of CAMHS resources, including human resources, service facilities and funding, with an insufficient number of CAMHS professionals and also students in training [16, 18, 11] (Table 1). The services available are affected by issues of understaffing and low capacity; thus, there was a need described for an upgrade of both both human resources and service facilities [16]. Currently, there are only five child psychiatrists in the whole country, compared with one traditional healer per 500 inhabitants to whom patients tend to turn instead [17, 21] (Table 1). Further task-sharing and in-service and pre-service training was described urgently needed, as this could help to integrate CAMHS into primary health care [22, 22].

#### Health information systems (overall policy and regulation)

This component refers to the overall policy and regulation of health systems and can be based on data at individual, health facility and population levels, and on public health surveillance. At the national level in Uganda the competency of health management information systems was found to be sufficient, but that was not the case at the district level. However, a stand-alone policy paper was drawn up for CAMHS in 2017, with an approach to increase availability and accessibility of quality CAMHS that aims to address the problems of underutilization of mental, neurological and substance health services by children and adolescents due to insensitivity of country health services, missed and mismanaged of early symptoms by parents, limited knowledge of mental health disorders and inadequate availability of quality CAMHS and also provides structure for the promotion of mental health of children and adolescent in different settings [13].

In addition, the health management information system was found to be insufficient for service planning, and resources were not distributed equally. Another study showed that stigma might contribute to the low priority given to mental health, and this in turn adds to the burden for children and adolescents and health care workers alike [17] (Table 1).

#### Access to essential medicines (output—availability and distribution of care)

Access to medication was found to be adequate and was offered at lower-level health centres as well. The availability of medications was regulated according to the Essential drug list for Uganda; however, this also depends on the presence of trained staff members who know how to use the drugs [18]. However, these services receive low priority and low levels of investment, contributing to further stigma and patients not seeking medication and treatment [16]. Levels of education of caregivers also play a role in stigma and attitudes associated with CAMHS: no or low levels of education could add to stigma but may also lead to inadequate incomes, limiting their ability to pay for drugs and treatment [16]. Parents or caregivers might also view and handle symptoms and mental issues differently from the formal health system and seek out other treatments such as those offered by traditional healers [17] (Table 1).

Generally, the studies found issues of poor public awareness and low willingness to seek treatment, as well as stigma against mental disorders. There could be a lack of motivation and knowledge to seek out treatment but also pressure from families, and drug costs could play a role [16]. Other barriers to treatment were family, community and individual attitudes, contributing to the burden of disease and a lack of help-seeking behaviour [16] (Table 1).

#### Health systems financing (input)

Services were underfunded by the government, and no donor funding was noted [18]. Funding was often geared towards mental health care in general and not centred on children and adolescents. However, the same was noted for all health services including primary health care, so in-kind support in the form of collaborations and refurbishment of infrastructure could be a way around this issue [18] (Table 1).

CAMHS is absent at lower-level health centres, hence the services are not yet offered in the primary health sector [11]. Moreover, the services available were found to be underfunded and centred around urban areas, meaning long travel distances and high costs for many in need [16]. Transport difficulties, transport time and costs can make such services inaccessible for children and adolescents in rural areas, who have few places nearby to seek help. The services offered in lower-level health centres were mostly centred on epilepsy, and psychosocial services were noted only in the national referral hospital [18] (Table 1). This is all connected to the lack of CAMHS-related legislation, policies and human resources [26].

#### Leadership and governance

One of the papers in the study, dating from 2015, found existing national mental health policies to be inadequate. These policies build on the United Nations Convention on the Right of the Child, the 1995 Constitution of the Republic of Uganda, the Mental Health Treatment Act 1964 and the Children Act 1996, but in 2015 there was no standalone policy on CAMHS itself. The laws and guidelines were acknowledged at the national level, but there was found to be little awareness at the district level [18]. However, since then, new policy guidelines for CAMHS have been designed, and were introduced in 2017 [13].

Several studies found there to be a lack of research and support for mental health needs, and this presents an enormous burden for which cost‐effective solutions are urgently needed [16, 11]. There is a lack of strategies and policies nationally, and those that exist tend to be focused on adult psychiatry and not on children and adolescents [18] (Table 1). This lack of attention to the planning of services and legislation adds to the burden of both patients and health care workers [26] (Table 1).

## Discussion

This paper provides an overview of services available for children and adolescents with mental health disorders in Uganda, their limitations and potential, and gives recommendations for the future. Twelve eligible studies were identified, five of which were qualitative studies and six quantitative, while one used a mixed-methods approach. CAMHS in Uganda requires improvement and needs to focus especially on vulnerable groups such as orphans, HIV/AIDS-affected youth and former child soldiers. These services show potential and there are multiple ways to address their limitations, such as collaboration with other sectors, integration into primary health care, reduction of stigma and a strengthened health workforce.

All studies reviewed showed that there is both an insufficient workforce and a lack of collaboration in the current CAMHS in Uganda. Multiple studies recommended increased collaboration to solve the problem of the shortage of health professionals. Also, due to the double burden of HIV/AIDS and mental distress, with HIV being widespread in the country, effective public health interventions and collaboration with the HIV/AIDS health care system are vital [16]. Another solution examined is for traditional healers and the biomedical sector to collaborate and potentially share the same referral systems, filling each other’s gaps. This solution has a lot of potential as some traditional healers are already using a number of biomedical methods, and due to their large numbers and presence in communities they might be seen as more approachable than health professionals [17]. However, some traditional healers are sceptical about the biomedical health care system and vice versa, so collaboration might not be so simple to achieve [11]. Other recommendations point to the implementation of CAMHS in primary health care, such as at lower-level health care centres. This implementation should aim to achieve increased access and effective utilization of mental health services, while also increasing awareness of CAMHS [21, 22].

There is a need for more effective dissemination of national policies, including the recently created standalone mental health policy for children and adolescents, but such policies need to be put into practice [13, 18]. For policies to succeed, the factors discussed above must be taken into account, and increased funding is needed [18].

International organizations and non-government organizations (NGOs) may also play a critical role in the planning and funding of mental health services. As mentioned earlier, only 1% of Uganda’s GDP is currently spent on mental health care, which is not sufficient [12] (even if there are other sources of funding such as AIDS or NGOs for mental health services which are not captured by the scientific publications included in this study). It is also important to encourage collaboration with stakeholders such as NGOs and policymakers, involving them in policy processes [18].

For the integration of services into primary health care, primary health care workers must be trained in child and adolescent mental health and there is also a need to improve training and introduce additional training for all mental health care professionals including nurses. This could improve identification and reporting of child and adolescent mental health cases by primary health care clinics. The health workers who are trained need to gain a perspective that includes both medical and public health-related factors, and services and the workforce need to be utilized to a maximum [22]. 

Lay workers and peers who are already present in the health and education systems need to be utilized and trained to provide mental health interventions. Recommendations call for both the training of primary health care professionals and the integration of CAMHS into existing sectors for better accessibility and increased resources [21, 22].

Access to medications could be improved by educating staff at lower-level health centres to clarify when and why to use such medications to help children and adolescents [18]. Due to the stigma associated with mental illness amongst children and adolescents, the attitudes of both society and individuals need to be changed if new interventions and increased access to care are to succeed. Young people must be able to recognize and respond appropriately to signs of distress, reduced functioning and other early signs of poor mental health [16, 19]. Therefore, stigma reduction strategies and awareness campaigns aimed at individuals, families and communities are recommended, such as asset-based interventions, i.e., child development accounts, which are critical in reducing risks associated with mental health challenges [19].

Finally, multiple studies suggest and explore new interventions to develop CAMHS, such as collaborations between multiple sectors. One example here is the double burden of HIV/AIDS and mental distress, where the primary health care sector should aim to increase access and effective utilization of services for patients considering all aspects of physical and mental health.

One way to increase the knowledge of primary health care workers in child and adolescent mental health is through the provision of in-service training, based on the mhGAP-IG. However, further studies are called for on task-sharing and integrating CAMHS into a larger sample of primary health care clinics, including a community mobilization component in the intervention to improve CAMHS attendance [21, 22]. This kind of training should be offered as both pre- and in-service training. Pre-service training is more cost-effective as staff do not have to take time off work, and students who have not yet finished their education can get an early introduction to the field [28, 29, 30, 31].

A different solution is further task-sharing with communities, implementing different types of treatment. In one study, community-implemented trauma therapy showed a reduction in PTSD and other mental health disorders, especially for the group receiving narrative exposure therapy [23]. Therefore, community-based interventions can be effective for children and adolescents affected by PTSD and other mental disorders. Involving members of the community to address mental health disorders has been shown to be effective and should be used further [23].

Another intervention often examined is economic empowerment. In one study, female participants receiving this intervention showed an improvement in mental health function over time. The intervention focused on females affected by HIV/AIDS in low-income settings, and economic empowerment took the form of peer mentorship and/or economic strengthening [19]. A similar intervention for children saw an improvement in their mental health functioning, such as reduced levels of hopelessness and depression. These results have further implications for public health programmes intended for long-term care of children living in resource-poor or AIDS-affected communities [24]. Another study agreed that there was evidence for the effects of economic empowerment interventions on children’s well-being including self-rated health and mental health functioning. This was also true for children affected by the double burden of HIV/AIDS and mental illness, and this calls for improved public policy and health programming for this group [24, 25].

Using the Frameworks for monitoring health systems performance designed by WHO, we have examined the current state of CAMHS in Uganda [27]. Our findings show potential for both development and new interventions, with the ultimate goal of increasing accessibility and distribution of these services. As previously described, children and adolescents are already vulnerable due to the changes they experience as they become older, but this is especially so in countries such as Uganda due to the high proportion of young people in the total population, diseases such as HIV/AIDS, conflicts, and poverty.

The challenges Uganda is facing in the area of child and adolescent mental health are not unique, and there is a widening mental health treatment gap for children and adolescents in all sub-Saharan Africa [32]. The region has few economic or human resources dedicated to the mental health of children and young people [32]. For example, a recent study from Tanzania aimed to identify, assimilate and analyze the literature on child and adolescent psychiatry highlighted the limited resources for child and adolescent mental health and the need for more research in the area [33].

Other regions with a high proportion of low- and middle-income countries for example the countries in the Middle East and Asia face similar challenges [34, 35].

For CAMHS in Uganda to improve, more research is needed, and development needs to build on existing resources, such as those of the primary health care sector.

### Strengths and limitations of the study

To our knowledge, this is the first scoping review to provide an overview of child and adolescent mental health services in Uganda. However, Certain limitations of this study should be considered in interpreting the findings. for example, only few studies had Uganda as their main focus, with majority focusing on multiple countries. Many of the studies only focused on specific groups such as those affected by HIV/AIDS and not the child and adolescent population in general. The limited number of studies available shows the need for further research on this topic, as mental health problems are a growing issue, and many children and adolescents are more vulnerable due to HIV/AIDS, war, orphanhood, and other challenges. Another limitation of this study is that no correlation could be made between the prevalence of child and adolescent mental health problems in the community and the proportion receiving treatment by CAMHS in Uganda. Furthermore, whilst there are key recommendations for further development of CAMHS in Uganda, the study could not determine a particular service model that clearly outlines the required manpower and available treatments including specific biomedical treatments, psychological therapies and psychosocial interventions. Whilst the study findings may be generalisable to other low and middle income countries, similar research in other comparable countries would be worthwhile before such conclusions could be reached.

### Implications of the study

The findings of this study have implications for new initiatives and policies. There is just recently developed a standalone mental health care plan or policy for children and adolescents alone, and the available services are scarce and centred in urban areas. Few of them are child- and adolescent-friendly, and vulnerable groups such as orphans and HIV/AIDS-affected youth should be given comprehensive treatment. There is also a need to reduce stigma and spread awareness through public campaigns and the integration of mental health into primary health care. Policymakers need to develop a stronger mental health workforce that can cater to the needs of children and adolescents, especially in a country like Uganda with its history of war, trauma and HIV/AIDS.

## Conclusion

As in other low and middle income countries, health system improvements and research driven changes need to be made in order to enhance CAMHS in Uganda. To address the lack of both human resources and facilities, collaboration with other sectors such as traditional healers and primary health care is necessary. One way to do this is to train health professionals with the help of the mhGAP-IG and using both in-service and pre-service training. Furthermore, mental health policies have already been developed, but they need to be modified to prioritize children and adolescents as well implementing the recent standalone policy of 2017. Moreover, to increase help-seeking behaviour, interventions need to focus on awareness and attitudes of society to help reduce stigma. Other interventions that could be implemented are community-based therapy and economic empowerment, targeting children and adolescents in low-resource settings and offering an alternative to formal CAMHS facilities. The main goal for CAMHS in Uganda is therefore to build on existing resources and facilities, increasing access, building a skilled workforce and extending collaboration, while reducing stigma and barriers to help-seeking.

## Data Availability

Not applicable.

## Abbreviations

CAMHS: Child and adolescent mental health services
GDP: Gross domestic product
PRISMA-ScR: Preferred Reporting Items for Systematic Review and M-Analyses Extension for Scoping Review
mhGAP: Mental Health Gap Action Programme
mhGAP-IG: Mental Health Gap Action Programme Intervention Guide
PTSD: Post-traumatic stress disorder
WHO: World Health Organization
WHO-AIMS: World Health Organization Assessment Instrument for Mental Health Systems
